# Pagetoid Reticulosis: A Rare Dermatologic Malignancy Presenting in a Middle-Aged Female

**DOI:** 10.7759/cureus.18524

**Published:** 2021-10-06

**Authors:** Samuel Stahly, Mitch Manway, Christine C Lin, Suporn Sukpraprut-Braaten

**Affiliations:** 1 Internal Medicine, Unity Health, Searcy, USA; 2 Dermatology, HonorHealth/Affiliated Dermatology, Scottsdale, USA; 3 Research, Unity Health, Searcy, USA

**Keywords:** dermatopathology, resistant dermatitis, clinical dermatology, cutaneous t cell lymphoma, pagetoid reticulosis

## Abstract

Pagetoid reticulosis is a rare form of cutaneous T cell lymphoma, a malignancy of T lymphocytes, that invades the skin leading to a multitude of dermatologic manifestations. Pagetoid reticulosis commonly presents as a localized slow-growing or indolently manifesting hyperkeratotic patch/plaque on the extremities that is confined to the epidermis. Diagnosis is confirmed via a skin biopsy of the affected area followed by a cytologic examination. Treatment typically entails topical corticosteroids, alkylating agents, and retinoids. With disseminated disease, a multidisciplinary approach involving chemotherapy and radiation is necessary. To raise awareness about pagetoid reticulosis, its diagnosis, and management, we report a case localized to the left forehead and earlobe of a 57-year-old female.

## Introduction

Cutaneous lymphomas are a rare subset that comprises 3.9% of all non-Hodgkin lymphomas [1]. Furthermore, cutaneous T cell lymphoma (CTCL) is a malignancy of T cells with an incidence of 9.6 cases per million people [1]. Pagetoid reticulosis represents a rare variant of CTCL characterized by groups of CD30+ malignant T cells invading the epidermis [2]. Lesions are typically localized to the extremities and can be effectively treated with topical agents [3]. However, a disseminated variant known as Ketron-Goodman disease [2] poses a challenge, requiring a multidisciplinary approach with systemic chemotherapy, topical agents, and biologics. The diagnosis is difficult due to the rarity of the condition, its indolent course, and its varying presentation. Here, we report the case of a 57-year-old female diagnosed with pagetoid reticulosis on the left ear and forehead.

## Case presentation

**Figure 1:**
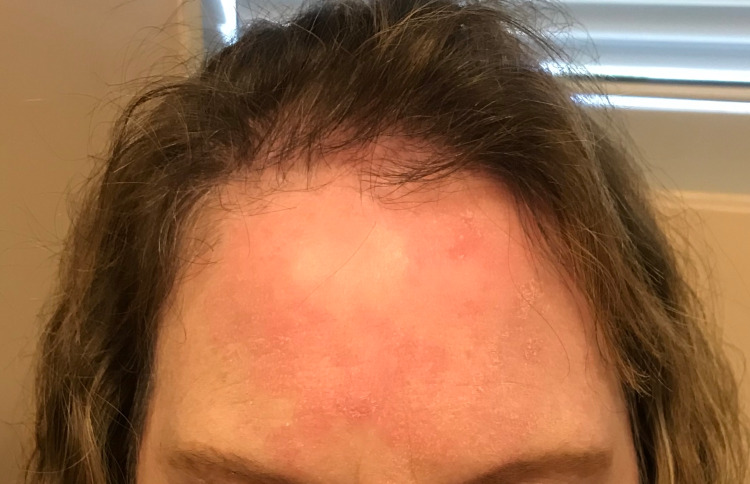
Pagetoid reticulosis presenting as a scaling hyperkeratotic plaque on the forehead of the patient.

A 57-year-old female presented to the dermatology clinic for evaluation of an erythematous rash on the left forehead and ear. The patient reported that the rash had developed two months prior. She stated that it had been asymptomatic and stable, and denied the use of any new cosmetics or cleansers. Her medical history was significant for allergic rhinitis, gastroesophageal reflux disease, and chronic ultraviolet exposure secondary to sun tanning. Incidentally, the patient had been self-treating the rash on her face with betamethasone which was originally prescribed for a distinct eczematous rash with no improvement. On physical examination, multiple discrete pink macules were noted over the left forehead with similar lesions over the left ear. Proposed differential diagnoses included cutaneous malignancy, tinea infection, and contact dermatitis. The helical lesion was cultured to rule out infection, and a shave biopsy was performed on the preauricular area to assess for cutaneous malignancy. Figure 1 shows the macular lesion on the left forehead of the patient.

Histologic examination of the lesion on the left preauricular area showed perivascular and periadnexal dermatitis, highly suspicious for a connective tissue disorder. Hematological examination including complete blood count (CBC), antinuclear antibody, and rheumatoid factor (RF) was unremarkable, except for RF which was elevated. Hydrocortisone cream 2.5% was prescribed for application to the affected areas, and a one-month follow-up was scheduled with an allergist to rule out contact dermatitis.

On follow-up, the patient reported no improvement of the rash and denied any associated pruritus. At this appointment, a diagnosis of seborrheic dermatitis was favored and treatment was initiated with ketoconazole 2% cream. The patient returned to the allergist one month later with no improvement in the rash following the trial of ketoconazole. She was then instructed to discontinue the ketoconazole and begin the application of pimecrolimus 1% cream to the affected areas. After this visit, the patient underwent allergy testing, but no relevant findings were noted. Due to the persistence of the rash, the patient requested reevaluation by dermatology.

**Figure 2:**
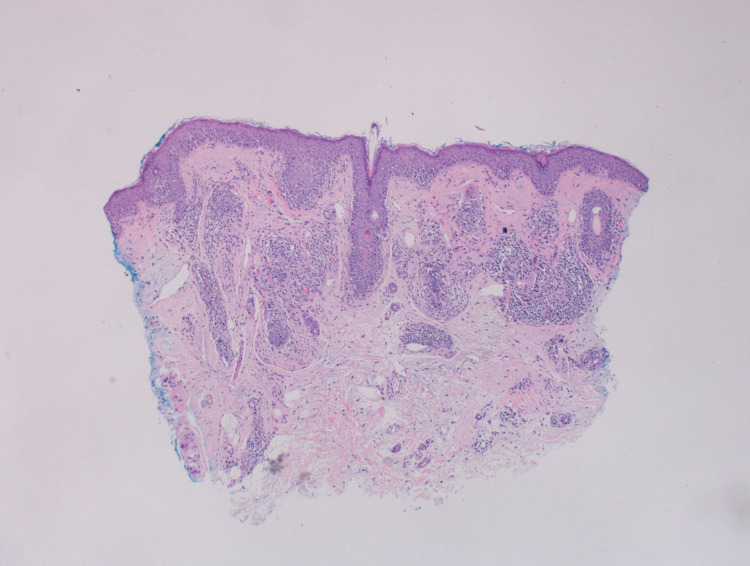
Pathology slide showing the full 3 mm punch biopsy obtained from the right forehead of the patient, which was positive for pagetoid reticulosis.

**Figure 3:**
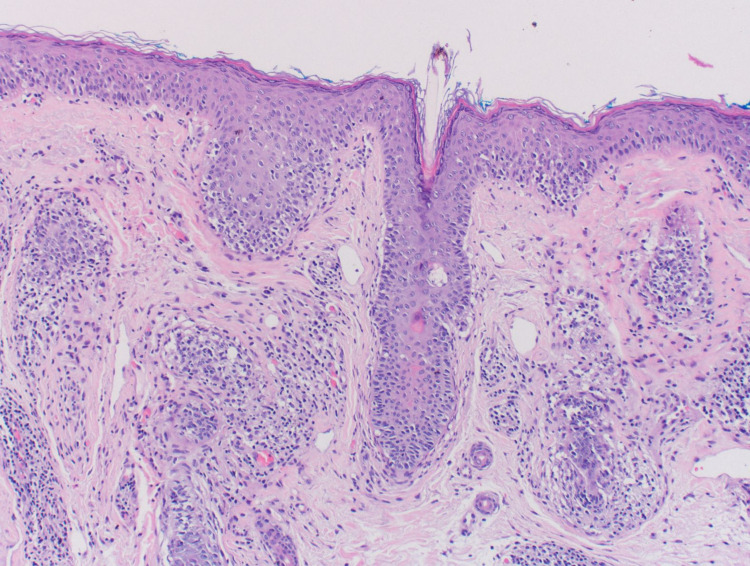
Biopsy specimen showing a periadnexal inflammatory infiltrate accompanied by dense intraepidermal mononuclear cell aggregates.

**Figure 4:**
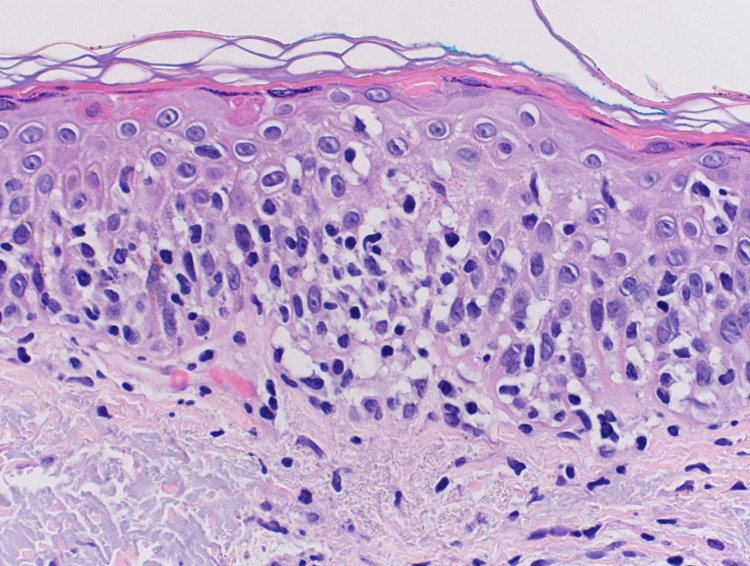
Higher-power view of the biopsy specimen showing the intraepidermal mononuclear cell aggregate with enlarged hyperchromatic nuclei, irregular nuclear borders, and abundant clear cytoplasm.

The patient consulted a dermatologist two weeks later. The rash did not show any improvement and was not associated with any new symptoms of burning or itching sensation. After a detailed examination of the forehead, it was decided that a 3 mm punch biopsy of the affected area was warranted. Figure 2 shows the full 3 mm punch biopsy taken from the left forehead. Figure 3 and Figure 4 show high-powered views of the biopsy specimen, significant for periadnexal inflammatory infiltrate consisting of intraepidermal mononuclear cell aggregates with enlarged hyperchromatic nuclei and irregular nuclear borders. These histologic findings were consistent with the diagnosis of pagetoid reticulosis. Following the diagnosis, a CBC, comprehensive metabolic panel, and T cell count were ordered, and the patient was instructed to apply Clobetasol 0.05% cream twice daily and return in one month for follow-up. Because the rash did not improve, the patient was referred to the Mayo Clinic where a trial of topical nitrogen mustard therapy was initiated without improvement of the rash. Furthermore, the patient noted spread to other areas of her body including the extremities and trunk. The patient’s current treatment regimen is light therapy three times per week with chlormethine topically.

## Discussion

Pagetoid reticulosis is an uncommon form of CTCL [4]. It is typically characterized as an erythematous and acanthotic patch on the skin that may involve deeper structures such as the blood vessels, lymph nodes, or viscera [5]. The condition can be easily mistaken for an inflammatory skin disorder, making the diagnosis challenging [5]. The rare variant, pagetoid reticulosis, consists of a localized form and a generalized form. The localized form, known as Woringer-Kolopp disease, presents as a single, slow-growing hyperkeratotic patch, whereas the generalized form, known as Ketron-Goodman disease, shows widespread involvement and is more aggressive [6].

The World Health Organization-European Organization for Research and Treatment of Cancer currently classifies Woringer-Kolopp disease as “a variant of mycosis fungoides characterized by the presence of localized patches or plaques with an intraepidermal proliferation of neoplastic T cells” [7]. The lesions are typically found on acral skin; however, this patient’s lesions were located on the ear and forehead. The differential diagnoses include psoriasis, Bowen’s disease, atopic dermatitis, and other forms of mycosis fungoides [8]. The indolent course of the condition makes it a challenge to differentiate it from other diseases and can prolong identification [8]. In this case, infectious, autoimmune, and allergic etiologies were pursued over the course of months before making the diagnosis. Therefore, pathologic examination of the lesions is required to confirm the presence of malignant T cells in the epidermis [9].

Dermatoscopy may help to narrow the differential diagnosis; however, biopsy followed by pathologic examination is needed to differentiate pagetoid reticulosis from other variants of CTCL [9]. A biopsy typically shows dense lymphoid aggregates confined to the epidermal layer of the skin. Immunologically, pagetoid reticulosis usually consists of CD4+ T cells, although rarer forms of CD8+ T cells and CD4-/CD8- T cells have been documented in the literature [10]. In addition, the condition is commonly CD30+ whereas the vast majority of other forms of CTCL are CD30- [7]. Clinicopathologic correlation is key in differentiating indolent versus aggressive forms of CTCL, which plays a significant role in determining the treatment of choice [9].

The majority of patients presenting with a localized form of CTCL have an excellent prognosis [3]. Lesions that are confined to the epidermis can be managed with topical agents such as corticosteroids, alkylating agents, or bexarotene, a topical retinoid [3]. In addition, targeted radiation therapy is another option for localized disease. Topical corticosteroids achieved complete remission in up to 63% of localized cases [11]. An alternative treatment for resistant lesions is nitrogen mustard cream, which prompted remission in 50-75% of cases [12]. In contrast, our patient saw no improvement with topical corticosteroids or nitrogen mustard therapy. Ultraviolet A and B radiation are additional therapies that are routinely employed for localized cases of CTCL [3]. Patients with a metastatic disease usually require a multidisciplinary approach, involving systemic chemotherapy and radiation combined with topical and biological agents [3]. Metastatic pagetoid reticulosis is more difficult to treat and has a higher mortality rate [3]. Determining the type of CTCL, its immunologic profile, and the stage of the disease is important for choosing a treatment that maximizes patient benefit while concomitantly minimizing side effects [3].

## Conclusions

The diagnosis of pagetoid reticulosis is challenging and treatment may take months to years before improvement is noted. We present this case to provide further evidence of the variable presentation and subtle onset of the disease. Due to minimal symptoms, many cases go undiagnosed for a prolonged period of time, as was the case with this patient. Fortunately, most cases remain localized to the epidermis and have a positive prognosis; however, there is potential for metastasis. As previously stated, early diagnosis of pagetoid reticulosis is key, as interventions are more effective when the disease is treated early. Physicians should be aware of this disease and its variable progression so that prompt diagnosis can be made and long-term sequelae can be minimized.

## References

[1] Chang TW, Weaver AL, Shanafelt TD (2017). Risk of cutaneous T-cell lymphoma in patients with chronic lymphocytic leukemia and other subtypes of non-Hodgkin lymphoma. Int J Dermatol.

[2] Haghighi B, Smoller BR, LeBoit PE, Warnke RA, Sander CA, Kohler S (2000). Pagetoid reticulosis (Woringer-Kolopp disease): an immunophenotypic, molecular, and clinicopathologic study. Mod Pathol.

[3] Wilcox RA (2011). Cutaneous T-cell lymphoma: 2011 update on diagnosis, risk-stratification, and management. Am J Hematol.

[4] Suzaki R, Kobayashi K, Ishizaki S, Fujibayashi M, Tanaka M (2010). Dermoscopic features of CD8-positive solitary pagetoid reticulosis on the left leg. Dermatol Res Pract.

[5] Fujii K (2018). New therapies and immunological findings in cutaneous T-cell lymphoma. Front Oncol.

[6] Carlesimo M, Tammaro A, Cox C (2009). A case of Ketron-Goodman disease. Case Rep Dermatol.

[7] Willemze R, Jaffe ES, Burg G (2005). WHO-EORTC classification for cutaneous lymphomas. Blood.

[8] Virmani P, Myskowski PL, Pulitzer M (2016). Unusual variants of mycosis fungoides. Diagn Histopathol (Oxf).

[9] Miedler JD, Kristjansson AK, Gould J, Tamburro J, Gilliam AC (2008). Pagetoid reticulosis in a 5-year-old boy. J Am Acad Dermatol.

[10] Larson K, Wick MR (2016). Pagetoid reticulosis: report of two cases and review of the literature. Dermatopathology (Basel).

[11] Zackheim HS, Kashani-Sabet M, Amin S (1998). Topical corticosteroids for mycosis fungoides. Experience in 79 patients. Arch Dermatol.

[12] Kim YH, Martinez G, Varghese A, Hoppe RT (2003). Topical nitrogen mustard in the management of mycosis fungoides: update of the Stanford experience. Arch Dermatol.

